# Two-color, one-photon uncaging of glutamate and GABA

**DOI:** 10.1371/journal.pone.0187732

**Published:** 2017-11-08

**Authors:** Stefan Passlick, Paul F. Kramer, Matthew T. Richers, John T. Williams, Graham C. R. Ellis-Davies

**Affiliations:** 1 Department of Neuroscience, Mount Sinai School of Medicine, New York, New York, United States of America; 2 Vollum Institute, Oregon Health and Sciences University, Portland, Oregon, United States of America; Thomas Jefferson University, UNITED STATES

## Abstract

Neuronal cells receive a variety of excitatory and inhibitory signals which they process to generate an output signal. In order to study the interaction between excitatory and inhibitory receptors with exogenously applied transmitters in the same preparation, two caging chromophores attached to glutamate and GABA were developed that were selectively photolyzed by different wavelengths of light. This technique has the advantage that the biologically inactive caged compound can be applied at equilibrium prior to the near instantaneous release of the transmitters. This method therefore mimics the kinetics of endogenously released transmitters that is otherwise not possible in brain slice preparations. Repeated photolysis with either of the two wavelengths resulted in GABA- or glutamate-induced activation of both ionotropic and metabotropic receptors to evoke reproducible currents. With these compounds, the interaction between inhibitory and excitatory receptors was examined using whole field photolysis.

## Introduction

The interaction between excitatory and inhibitory signals is the basis of neuronal computation. Our knowledge of the physiology of the underlying receptors is largely based on different techniques of direct agonist application to the receptors. However, ultra-fast application, which is necessary to generate physiologically relevant responses, is difficult to achieve in brain slices. More importantly, these techniques do not allow the application of two different agonists, i.e. excitatory and inhibitory agonists, to the same preparation at the same time to study their interaction. Single photon (1P) uncaging of compounds in brain slices[1] has been instrumental in obtaining pharmacological and kinetic information that is not possible with other forms of exogenous application[2,3]. Until recently, the use of two caged compounds that allow wavelength-selective activation of different receptors to study their interaction has not been possible. A handful of two-color uncaging studies of neurons in brain slices have appeared recently[4,5,6], however none of these use 1P excitation in both optical channels[7]. The key feature for such optically independent photolysis of two caged compounds is the lack of excitation of the longer wavelength chromophore by light used to excite the shorter wavelength chromophore[7]. The first report of an attempt to use two-color photolysis was in fact with dual 1P activation. This study used 254 nm and 420 nm light for selective photoremoval (i.e. uncaging) of two photochemical protecting groups [8]. The photolysis ratio of the long wavelength chromophore at these wavelengths was 15/85, thus two-color uncaging had only modest wavelength selectivity. Subsequent applications of wavelength-selective, 1P uncaging in a biological context used 355–380 nm and 420–440 nm light. In these reports only sequential uncaging was possible, with the long wavelength probe being photolyzed in its entirety first, before the second, short wavelength compound was activated [9,10]. Wavelength-selective 1P uncaging of glutamate and GABA on hippocampal neurons has been reported using 250–260 nm and 405 nm light [11]. However, in addition to requiring high-energy UV-C light, which is not compatible with common microscope objectives, the activation kinetics of the receptor-dependent currents were distinctly slow. Thus, there is still a need for two optical probes that can be cleanly photolyzed with two colors in the visible range such that each wavelength can be applied to neurons in brain slices so as to evoke individual effects. The work described here addresses this problem.

We have synthesized a long wavelength absorbing caged GABA probe (called DEAC454-GABA) that absorbs minimally in the region traditionally used for uncaging probes optimized for near-UV photolysis (340–370 nm range). Thus, when combined with a short wavelength absorbing caged glutamate, photolysis of these compounds resulted in the selective activation of glutamate and GABA receptors. In hippocampal CA1 cells, induction of action potentials evoked by glutamate was transiently blocked by prior photolysis of caged GABA. In dopamine neurons, the inhibition of spontaneous action potentials was induced by photolysis of GABA, which resulted in the activation of both GABA-A and GABA-B receptors. Likewise, photolysis of glutamate on dopamine neurons resulted in the activation of both AMPA and metabotropic glutamate receptors (mGluRs). Thus, the wavelength selectivity offers an ideal way to use 1P activation to examine mechanisms that underlie the modulation of neuronal activity.

## Results

### Synthesis and characterization of caged GABA

**Fig 1:**
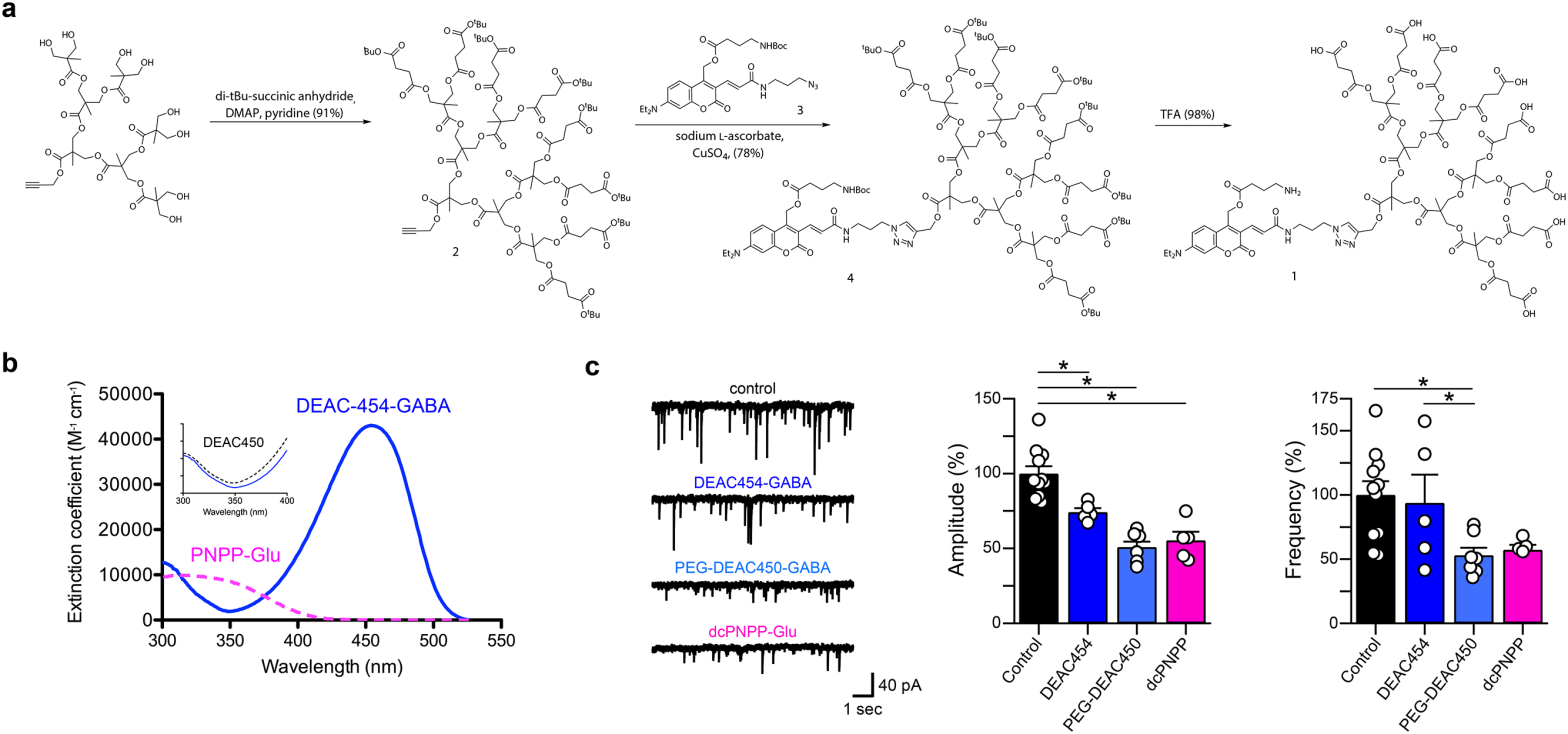
Synthesis, pharmacology and absorption spectrum of DEAC454-GABA. (a) Synthetic scheme for DEAC454-GABA (**1**). (b) Absolute absorption spectra of DEAC454-GABA (blue lines) and dcPNPP-Glu (violet dashed). Inset: normalized absorption spectra of DEAC454-GABA and PEG-DEAC450-GABA (black dashed) showing their relative minima in the near-UV. (c) Left, representative recordings of mIPSCs from hippocampal CA1 neurons under control conditions and in the presence of 28–31 μM DEAC454-GABA, 29–31 μM PEG-DEAC450-GABA or 290 μM dcPNPP-Glu. Right, summaries of the relative effects of DEAC454-GABA (n = 5 cells), PEG-DEAC450-GABA (n = 7 cells) and dcPNPP-Glu (n = 5 cells) on mIPSC amplitude and frequency compared to control recordings (n = 11 cells). For each cell, >200 events were analyzed. All compounds significantly reduced the amplitude while the frequency was only affected by PEG-DEAC450-GABA (* p < 0.05; One-Way ANOVA with post-hoc Tukey test). Recordings were made in the presence of TTX (1 μM), AMPA (CNQX, 10 μM) and NMDA receptor (DL-AP5, 100 μM) antagonists.

The synthesis of the 7-diethylaminocoumarin derivative DEAC454-GABA (**1**) is shown in Fig 1a. Dendrimer **2** was synthesized by reaction of *t*-butyl-succinic anhydride with the alcohol terminal groups of a known third-generation 2,2-bismethylolpropionic acid dendrimer [12]. A copper-catalyzed alkyne-azide “click” cycloaddition was used to conjugate **2** and **3** to afford **4** in 78% yield. The buffer soluble, octacarboxylic DEAC454-GABA (**1**) was obtained by treatment of **4** with trifluoroacetic acid in dichloromethane. In contrast to PEG-DEAC450-GABA and dcPNPP-Glu, this probe could be used with living cells without purification by HPLC.

The optical properties of DEAC454-GABA were different than the previously-developed PEG-DEAC450-GABA [13]. Extended π-electron derivatives of DEAC [14,15] and related coumarins [16] absorb light efficiently, and in the case of PEG-DEAC450 and DEAC454 the ε is 43,000 M^-1^ cm^-1^. Interestingly, close examination of the absorption spectrum revealed that embedding DEAC450 in the polyanionic dendrimer caused subtle changes in its optical properties. The λ_max_ was shifted to 454 nm and, though the λ_min_ was unchanged in position, its value was decreased by 50% compared to PEG-DEAC450-GABA (Fig 1b). The quantum yield of DEAC454 photolysis was also reduced, when compared with PEG-DEAC450-GABA [13], from 0.39 to 0.23.

The polyanionic dendrimer also changed the pharmacological properties of the compound. Caged glutamate and GABA probes are antagonistic towards GABA-A receptors. Initially part of our development of a new technology we call “cloaked caged compounds”[17], we found that the GABA-A receptor antagonism of DEAC454-GABA was modestly reduced compared to PEG-DEAC450-GABA[13] as shown by analysis of mIPSCs (miniature inhibitory post-synaptic currents) at set concentrations of our caged compounds (Fig 1c). The inhibition of GABA-A receptors limits the use of this compound at the high concentrations (0.1–10 mM) required in experiments involving two-photon (2P) induced photolysis [7,18,19]. However, the concentration range that is used for 1P uncaging is substantially lower, suggesting that DEAC454-GABA could be effective [7]. With the recent development of the caged glutamate probe dcPNPP-Glu [20] with a λ_max_ close to the λ_min_ of DEAC454-GABA (Fig 1b), these compounds could form a complimentary optical pair for wavelength-selective 1P uncaging. Thus, orthogonal two-color, 1P uncaging of GABA and glutamate could be possible.

### Functional optical cross-talk

**Fig 2:**
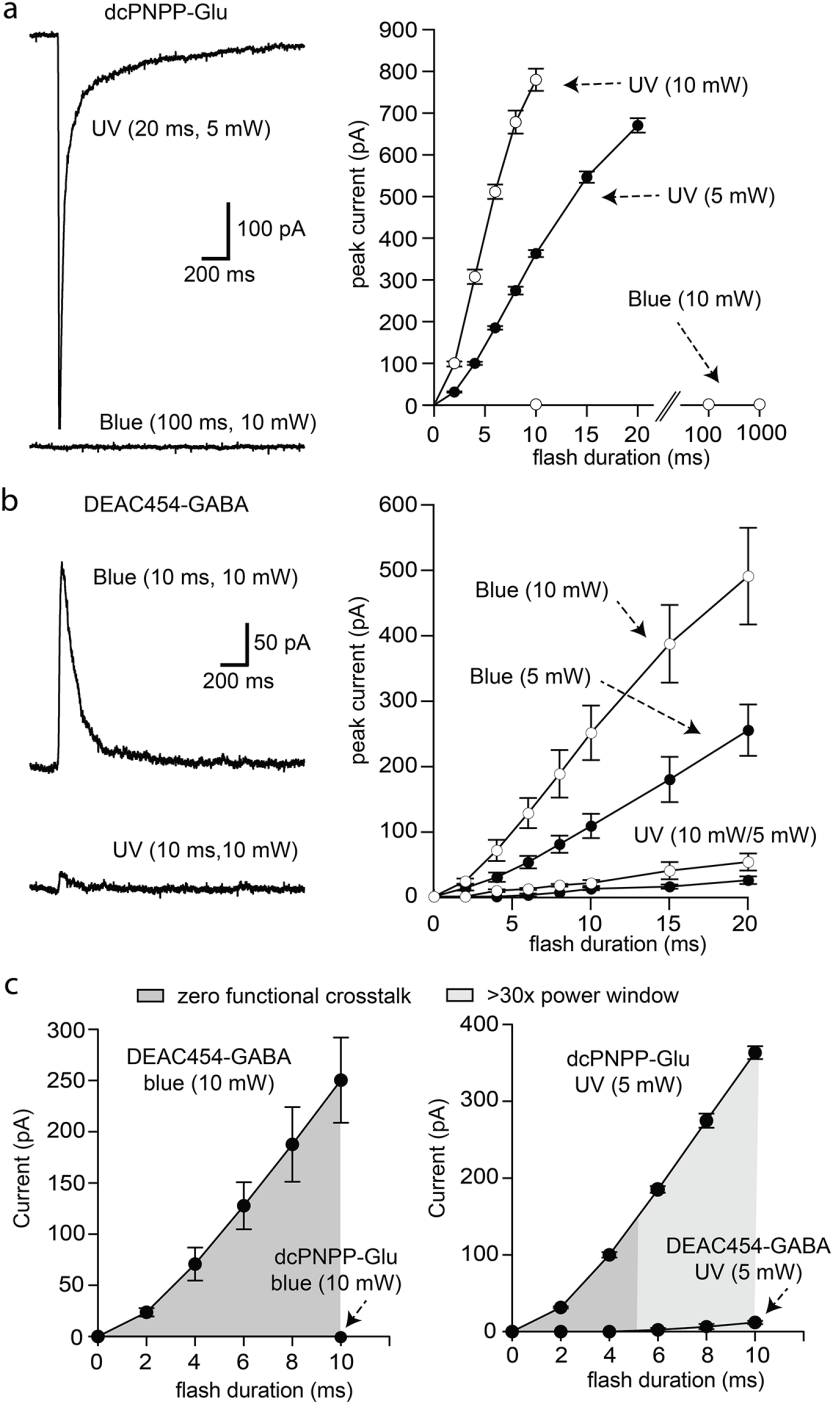
Wavelength-selective photostimulation of AMPA and GABA-A receptors in hippocampal CA1 neurons. (a) Currents induced by near-UV and blue light in a recirculating solution of dcPNPP-Glu. Left, example traces of currents induced by near-UV (top) and blue (bottom) light. Right, increasing the duration of near-UV light flashes increased the amplitude of the inward current that was power dependent (n = 3 cells). Blue light was ineffective at all flash durations. (b) Currents induced by blue and near-UV light in a recirculating solution of DEAC454-GABA. Left, example traces of currents induced by blue (top) and near-UV (bottom) light. Right, Summary of results using different duration flashes of blue and near-UV light at two different powers (n = 3 cells). (c) Plots illustrating the power window of dcPNPP-Glu and DEAC454-GABA of data shown in (a/b). Left, there is no functional cross-talk between the two caged compounds with the use of blue light. Right, there is minimal functional cross-talk between the two caged compounds with the use of near-UV light. Recordings were made in the presence of TTX (1 μM), NMDA (DL-AP5, 100 μM) and GABA-B receptor (CGP-55845, 3 μM) and mGluR (JNJ-16259685, 1 μM; MPEP, 3 μM; LY341495, 30 nM) antagonists. For all cells, a single data point was collected at each flash duration and the data were averaged across cells.

First, the functional optical cross-talk was determined for both caged compounds separately in neurons of the hippocampus and substantia nigra in acute brain slices. Near-UV light (365 nm) was used for photolysis of caged glutamate to evoke AMPA receptor currents in pyramidal neurons of the hippocampal CA1 region. A solution of dcPNPP-Glu (330 μM) was re-circulated [20]. Flashes of near-UV light (365 nm, 5 and 10 mW) evoked inward currents that were dependent on the duration and power of the flash (Fig 2a). No current was evoked when blue light (470 nm, 10 mW, 10–1000 ms) was used (Fig 2a). This result was expected due to the lack of absorbance of the short wavelength caging chromophore dcPNPP in the blue region (Fig 1b).

The next step was to examine the selective uncaging of the long wavelength cage DEAC454-GABA under similar conditions. In a solution containing DEAC454-GABA (27 μM), flashes of 470 nm light evoked GABA-A receptor-mediated outward currents that increased with duration and/or power of the flash (Fig 2b). More importantly, 365 nm light evoked no current above the noise (i.e. no functional cross-talk) with 5 mW and up to 4 ms duration (Fig 2b and 2c). Even flashes that were 10 ms in duration only produced a current of up to 12 pA whereas the same dosage of near-UV light induced large glutamate currents yielding a power window of >30-fold (Fig 2c). The amplitude of ionotropic receptor currents in whole-cell recordings is determined by the reversal potential for the underlying ion. To examine the limits of our approach, experiments were conducted with an increased driving force for chloride (80 mV from reversal potential vs. 60 mV from reversal potential for glutamate currents).

**Fig 3:**
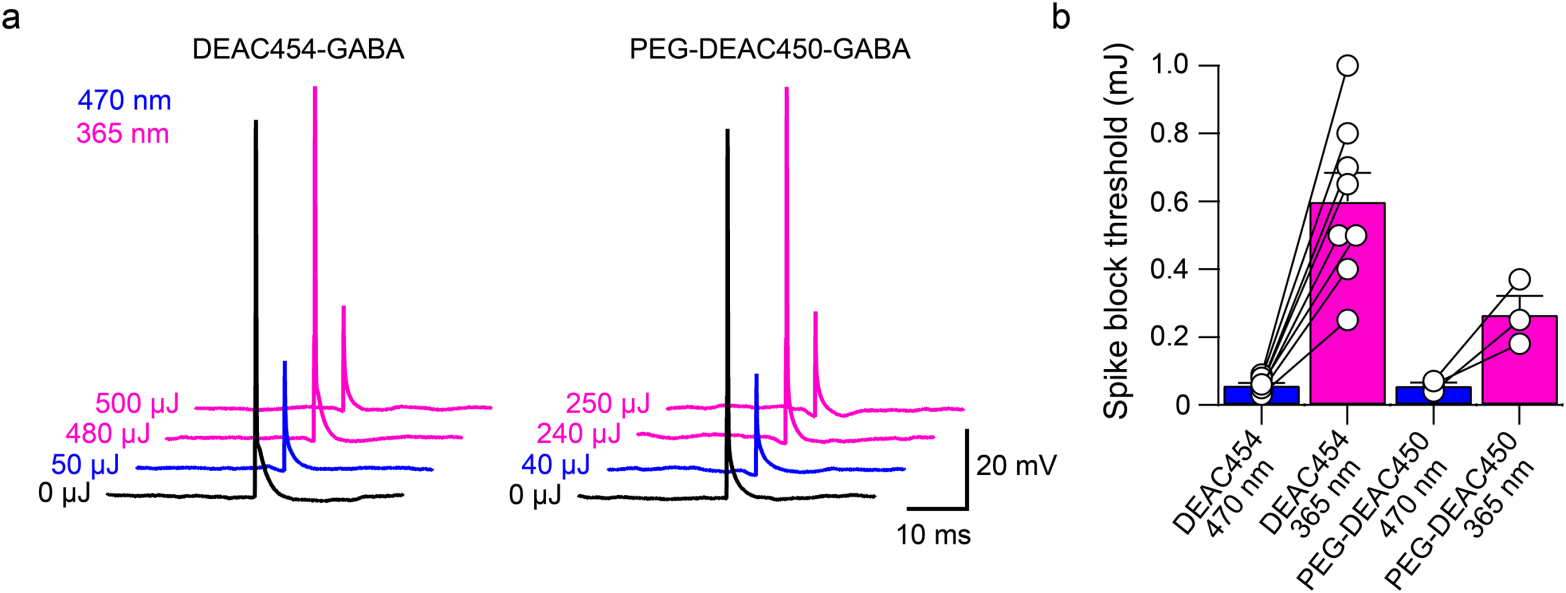
Block of current injection-evoked action potentials by DEAC454-GABA vs. PEG-DEAC450-GABA uncaging with blue or high intensity near-UV light in hippocampal CA1 neurons. (a) Example traces demonstrating blockade of current injection-evoked action potentials by DEAC454-GABA (left) and PEG-DEAC450-GABA (right) uncaging with 470 and 365 nm light in hippocampal CA1 neurons. Black: no uncaging; blue: 470 nm, violet: 365 nm. (b) Summarized results comparing the power threshold for action potential blockade using blue or near-UV light for DEAC454-GABA (n = 8 cells) and PEG-DEAC450-GABA (n = 3 cells). For all cells, current-injections were paired with increasing energy of light. The light energy threshold to block the action potential was averaged across cells. Recordings were made in the presence of AMPA (CNQX, 10 μM), NMDA (DL-AP5, 100 μM) and GABA-B receptor (CGP-55845, 3 μM) and mGluR (JNJ-16259685, 1 μM; MPEP, 3 μM; LY341495, 30 nM) antagonists.

To directly assess the cross-talk of near-UV light activating the long wavelength cage DEAC454-GABA, action potentials were elicited in hippocampal CA1 neurons by current injection from a patch pipette and were blocked by uncaging DEAC454-GABA with 470 and 365 nm light, respectively (Fig 3). When using 365 nm light, the power/duration of the flash had to be increased by about 10x to block action potential generation as compared to 470 nm light (Fig 3b). Importantly, when doing the same experiment with PEG-DEAC450-GABA, only 5x more 365 nm compared to 470 nm light was necessary to block the action potential (Fig 3b). This is consistent with the 50% reduced absorbance of DEAC454- vs. PEG-DEAC450-GABA in the short wavelength range (Fig 1b). Note, an increase in duration of the flash (25–100 ms) was necessary because power in the near-UV range was at the maximum. In addition, significant inner filtering at 470 nm occurs with 27–29 μM DEAC454-GABA compared to 365 nm, so the cross-talk is probably over estimated.

**Fig 4:**
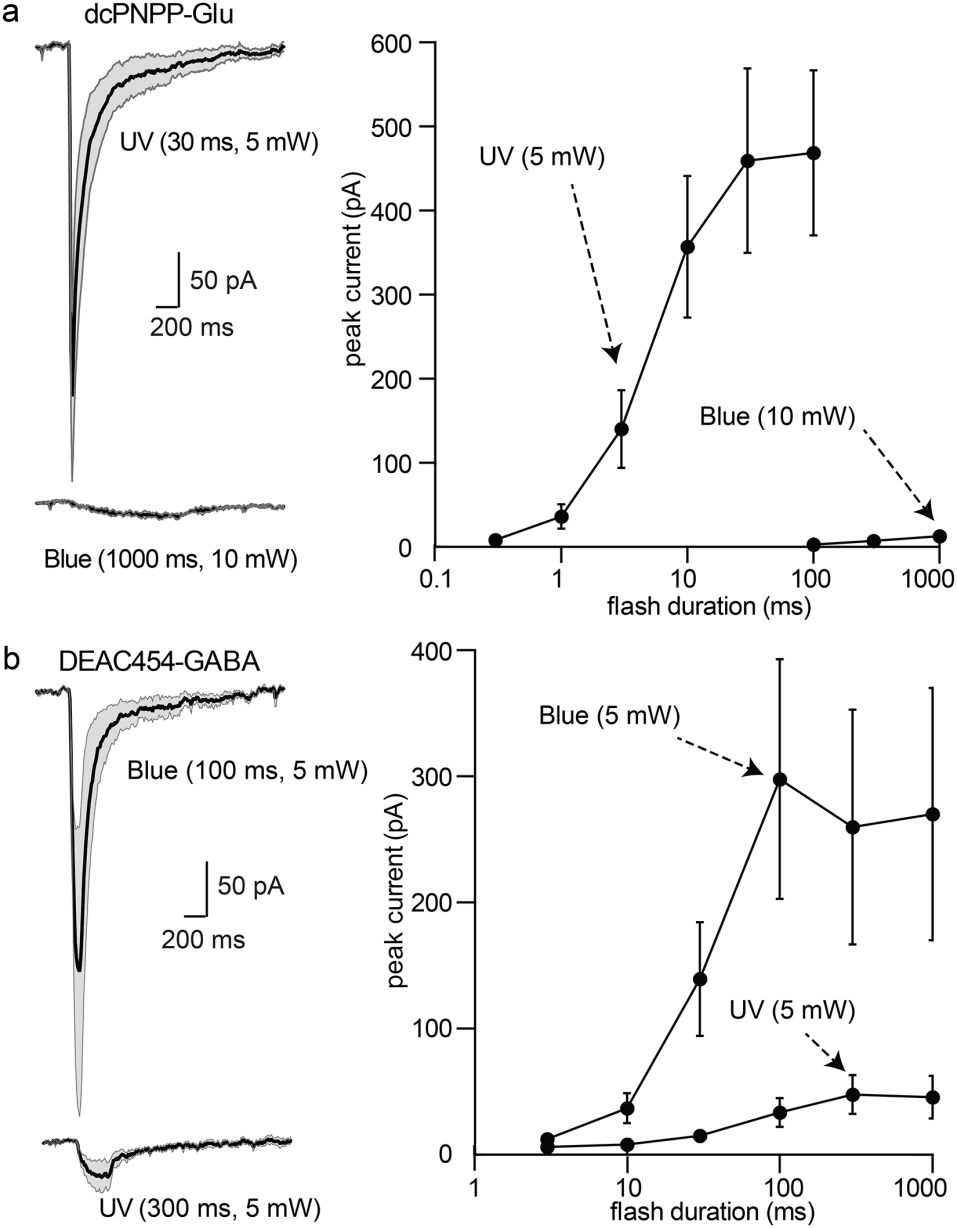
Wavelength-selective photostimulation of AMPA and GABA-A receptors in dopamine neurons of the substantia nigra. (a) Left, summary of inward currents induced by photolysis of dcPNPP-Glu by near-UV and blue light. Solid black line indicates the mean current and the gray area indicates the 95% confidence limits. Right, summary of the increase in inward current induced by longer duration flashes (n = 9 cells). (b) Left, summary of inward currents induced by photolysis of DEAC454-GABA by blue and near-UV light. Right, summary of the increase in current induced by longer pulses of light (n = 9 cells). Recordings were made in the presence of TTX (1 μM), NMDA (CPP, 10 μM), D2 (sulpiride, 2 μM) and GABA-B receptor (CGP-55845a, 500 nM) and mGluR (JNJ-16259685, 1 μM; MPEP, 3 μM) antagonists. For all cells, a single data point was collected at each flash duration and the data were averaged across cells.

In brain slices containing dopamine neurons in the substantia nigra, photolysis of dcPNPP-Glu (200–350 μM) with 365 nm light at 5 mW for periods of 1–100 ms produced currents of 30–350 pA that were dependent on the duration of the flash (Fig 4a). Flashes of blue light (450 nm) of up to 1000 ms resulted in currents less than 20 pA (Fig 4a). When DEAC454-GABA (30 μM) was re-circulated, blue light irradiation (5 mW, 3–300 ms duration, Fig 4b) evoked GABA-A receptor currents. The amplitude of the GABA-A receptor current peaked with a flash duration of 30 ms. When near-UV light was used, no current was activated with flash durations up to 30 ms and at 1000 ms a current of about 30 pA was evoked. The larger power window compared to hippocampal CA1 neurons is a result of the driving force which was 60 mV for excitation and 45 mV for inhibition highlighting the importance of the experimental conditions for assessment of optical cross-talk.

**Fig 5:**
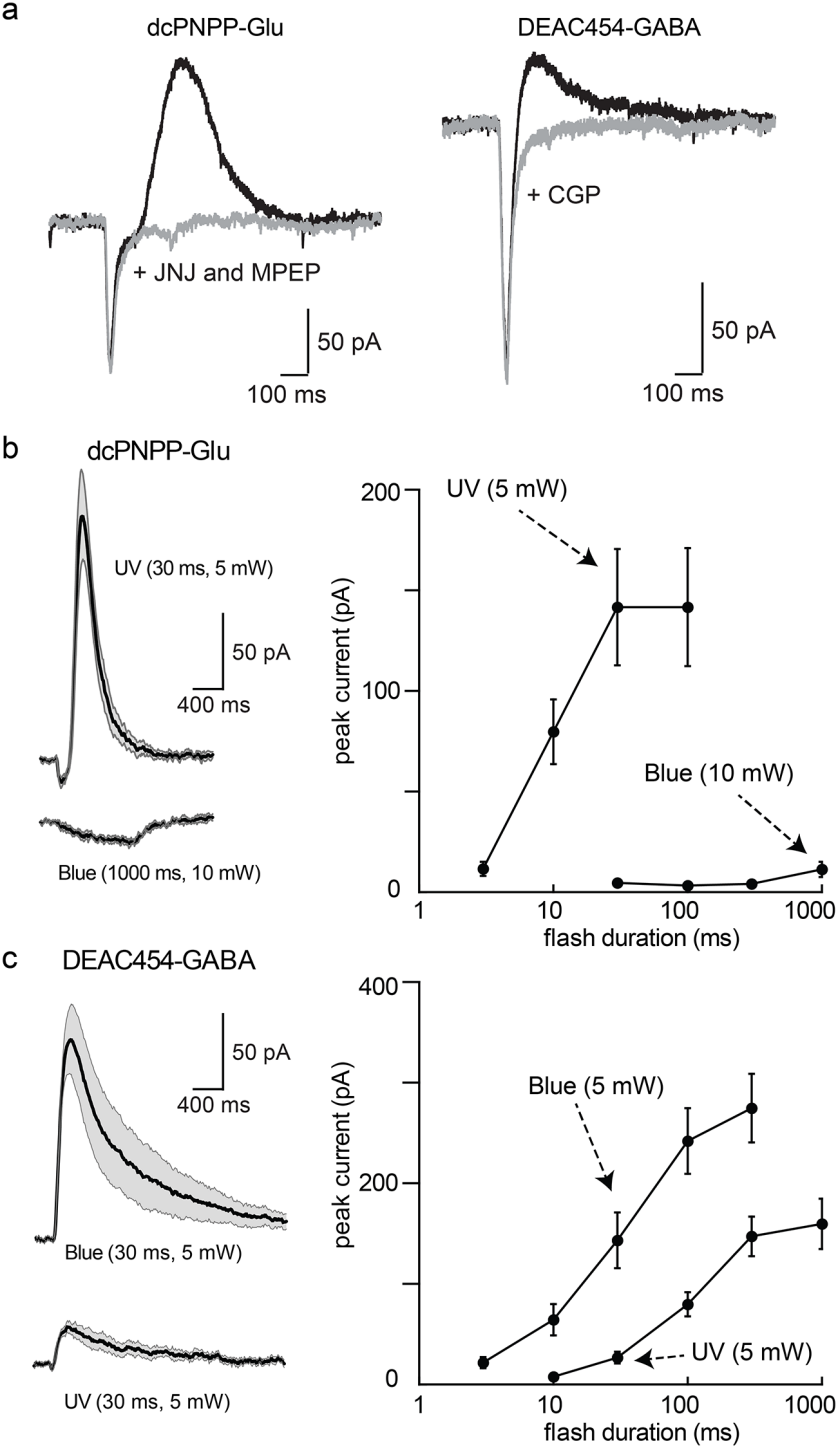
Wavelength-selective photostimulation of mGluRs and GABA-B receptors in dopamine neurons of the substantia nigra. (a) Left, example traces of the currents induced by photolysis of dcPNPP-Glu in control and following treatment with the mGluR1/5 antagonists JNJ16259685 (1 μM) and MPEP (3 μM). Right, example traces of currents induced by photolysis of DEAC454-GABA in control and following treatment with the GABA-B receptor antagonist CGP-55845a (500 nM). Note, the fast ionotropic inward current was unaffected in both cases. (b) Left, summary of currents induced by photolysis of dcPNPP-Glu by near-UV and blue light. Solid black line indicates the mean current and the gray area indicates the standard error of the mean. Right, summary of the increase in outward current induced by longer duration flashes (n = 8 cells). Blue light applied for longer than 100 ms resulted in an inward current that was also blocked by mGluR antagonists (not shown). (c) Left, summary of outward current induced by photolysis of DEAC454-GABA by blue and near-UV light. Solid black line indicates the mean current and the gray area indicates the standard error of the mean. Right, summary of the increase in current induced by longer pulses of light (n = 8 cells). Recordings were made in the presence of NMDA (CPP, 10 μM) and D2 receptor (sulpiride, 2 μM) antagonists. Those in (b/c) also contained GABA-A (picrotoxin, 10 μM) and AMPA receptor (CNQX, 10 μM) antagonists. For all cells, a single data point was collected at each flash duration and the data were averaged across cells.

Next, the activation of GABA-B and mGluR1/5 receptors was examined in dopamine neurons. When dcPNPP-Glu was re-circulated, flashes of near-UV light resulted in an inward (AMPA receptor) current followed by a slower outward current mediated by the calcium activated potassium conductance (SK, Fig 5a). The SK current could be blocked by mGluR1/5 antagonists, while the inward current remained unchanged (Fig 5a). Photolysis of DEAC454-GABA resulted in an inward (GABA-A receptor) current followed by an outward current mediated by GABA-B receptors. The outward current, mediated by the G protein-coupled inwardly rectifying potassium conductance (GIRK) was blocked by the GABA-B receptor antagonist, CGP-55845 (Fig 5a).

To examine the functional cross-talk of two-color uncaging for metabotropic receptors, AMPA and GABA-A receptors were blocked. In the presence of dcPNPP-Glu, flashes of near-UV light activated mGluRs to induce a SK-dependent outward current that was dependent on the duration of the light pulse (Fig 5b). No current was evoked with short flashes of blue light (Fig 5b). Longer flashes (>300 ms) resulted in a small mGluR-dependent inward current that is only seen with iontophoretic applications >500 ms and bath application of agonist. When DEAC454-GABA was re-circulated, flashes of blue light (5 mW, 3–300 ms) resulted in the activation of the GIRK current mediated by GABA-B receptors (Fig 5c). Unlike the results obtained with the activation of GABA-A receptors, a substantial GABA-B dependent current was evoked with the application of near-UV light, although longer duration flashes were required (Fig 5c). This likely results from the increased sensitivity and amplification of downstream G protein signaling induced by GABA-B receptors.

Taken together, the results from the hippocampus and substantia nigra show that, using appropriate concentrations of caged glutamate and GABA, there is a substantial power window where there is functionally zero optical cross-talk for each probe with 365 nm and 450–470 nm light.

### Co-application of DEAC454-GABA and dcPNPP-Glu

**Fig 6:**
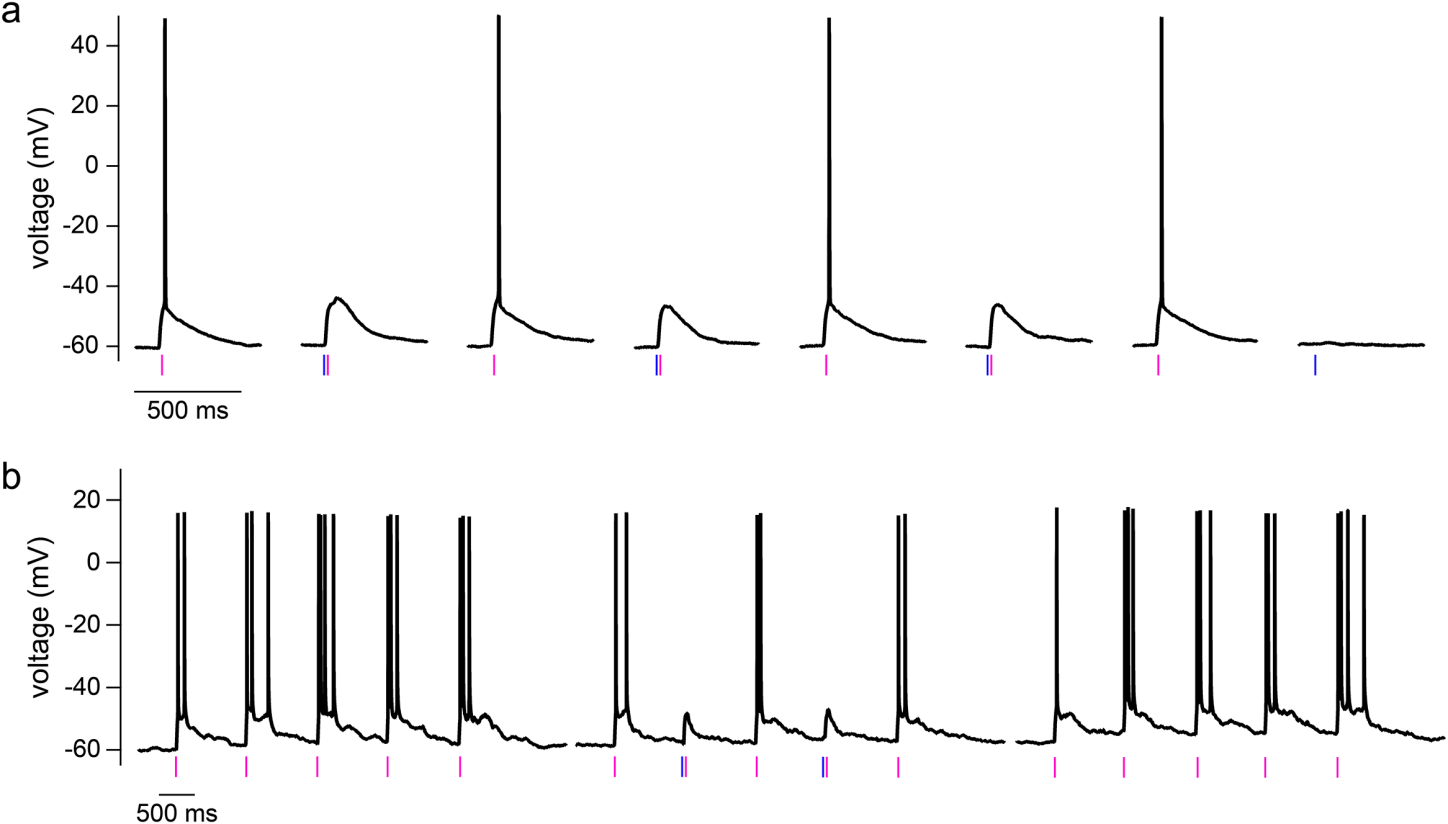
Arbitrarily ordered two-color uncaging of glutamate and GABA on hippocampal CA1 neurons. (a) In presence of dcPNPP-Glu and DEAC454-GABA, irradiation with 365 nm light (5 mW, 6 ms) evoked single action potentials. Preceding this stimulus with 470 nm light irradiation (10 mW, 1 ms) reversibly blocked the action potential. The 470 nm light stimulus alone did not evoke a detectable change in membrane potential (shunting inhibition). (b) 365 nm light irradiation (5 mW, 4 ms) at 1 Hz evoked multiple action potentials (left). Preceding the 2^nd^ and 4^th^ stimulus with 470 nm light irradiation (10 mW, 12 ms) reversibly blocked the action potentials selectively on these stimuli (middle). Removal of the blue flashes restored reliable action potential responses (right). DEAC454-GABA and dcPNPP-Glu were bath-applied at 27 μM and 310 μM, respectively. Recordings were made in the presence of NMDA (DL-AP5, 100 μM) and GABA-B receptor (CGP-55845, 3 μM) and mGluR (JNJ-16259685, 1 μM; MPEP, 3 μM; LY341495, 30 nM) antagonists.

Having established power domains for selective photoactivation of neurotransmitter receptors when each caged compound was applied separately to neurons, conditions for bidirectional manipulation of membrane potential were explored with co-application of DEAC454-GABA (27 μM) and dcPNPP-Glu (310 μM) to brain slices. In hippocampal CA1 neurons, near-UV light alone evoked action potentials by photolysis of caged glutamate (Fig 6a). When the same stimulus was preceded with blue light, the action potential was blocked by photolysis of caged GABA (Fig 6a). Experiments with one or both light flashes were carried out at 15 s intervals. Since experiments were conducted close to the reversal potential of chloride, 470 nm light alone did not elicit a significant change in membrane potential, a phenomenon known as shunting inhibition. Uncaging experiments were also performed at 1 Hz, resulting in trains of action potentials (Fig 6b). When blue light was co-applied at the 2^nd^ and 4^th^ trial, the action potentials were blocked (Fig 6b). Finally, repeated pulses of near-UV light alone resulted in a train of action potentials demonstrating full reversibility (Fig 6b). It is notable that short wavelength light applied multiple times before applying the long wavelength did not significantly deplete the long wavelength caged compound. The two examples in Fig 6 show that a stronger stimulus might induce a single action potential in one cell (Fig 6a) while a milder stimulus might induce a burst of action potentials in another cell (Fig 6b). Several factors including location of stimulus relative to cell, depth of cell in brain slice, glutamate reuptake, and membrane potential or cell health may influence the cellular response to a given stimulus. By carefully titrating the light energy applied, a variety of cellular responses may be studied in the same cell.

**Fig 7:**
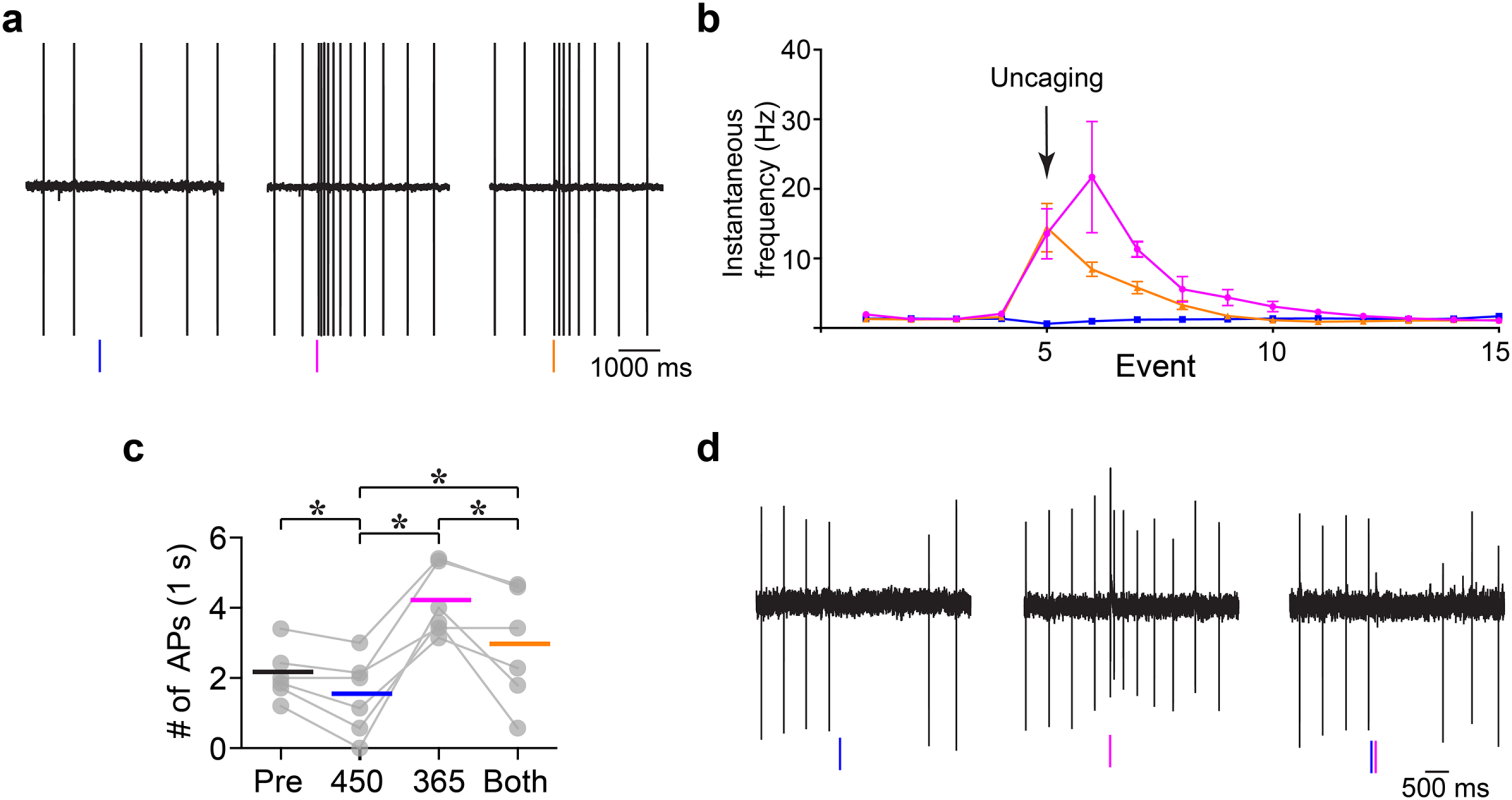
Arbitrarily ordered two-color uncaging of glutamate and GABA on dopamine neurons of the substantia nigra. (a) Example of an experiment showing wavelength-dependent changes in spontaneous firing by activation of AMPA and GABA-A receptors. Left, the frequency of action potentials was decreased by photolysis of DEAC454-GABA with 450 nm light (100 ms, middle) and increased by photolysis of dcPNPP-Glu using 365 nm light (10 ms, middle). Right, simultaneous photolysis resulted in a decrease in the excitation induced by dcPNPP-Glu by activation of GABA-A receptors. Experiments were conducted in cell-attached mode. (b) Summary of change in instantaneous frequency in response to glutamate and GABA uncaging as shown in (a) (n = 6 cells, 3 recordings per cell per condition). Blue line: events when 450 nm light was flashed. Violet line: events when 365 nm light was flashed. Orange line: events when both wavelengths were flashed simultaneously. (c) Summary of change in number of action potentials in response to glutamate and GABA uncaging in 1 s following photolysis as shown in (a) (n = 6 cells, 5–7 recordings per cell per condition). Bars indicate means. Each group represents number of action potentials. Pre: 1 s prior to 450 nm LED flash, 450: 1 s after 450 nm LED, 365: 1 s after 365 nm LED, and Both: 1 s after 450 and 365 nm LEDs flashed simultaneously. A repeated measures two-way ANOVA was used with a post-hoc Tukey test to examine multiple comparisons (all comparisons *P* < 0.001). (d) Example of an experiment in cell-attached mode showing wavelength-dependent changes in spontaneous firing by activation of AMPA and GABA-B receptors. Left, the frequency of action potentials was decreased by photolysis of DEAC454-GABA using 450 nm light and increased by photolysis of dcPNPP-Glu with 365 nm light. Right, simultaneous photolysis resulted in a decrease in the excitation induced by dcPNPP-Glu by activation of GABA-B receptors. In experiments (a-c), DEAC454-GABA and dcPNPP-Glu were bath-applied at 30 μM and 300 μM, respectively, together with NMDA (CPP, 10 μM), D2 (sulpiride, 2 μM) and GABA-B receptor (CGP-55845a, 500 nM) and mGluR (JNJ-16259685, 1 μM; MPEP, 3 μM) antagonists. In (d), DEAC454-GABA (25 μM) and dcPNPP-Glu (200 μM) were bath-applied together with NMDA (CPP, 10 μM), D2 (sulpiride, 2 μM) and GABA-A receptor (picrotoxin, 10 μM) and mGluR (JNJ-16259685, 1 μM; MPEP, 3 μM) antagonists.

Finally, two-color uncaging in dopamine neurons was examined. Because dopamine neurons fire action potentials in a tonic pacemaker pattern, activation by caged glutamate and inhibition by caged GABA could both be examined (Fig 7). Flashes of 470 nm light induced a pause in spiking while 365 nm light increased the firing rate. When both wavelengths were applied simultaneously an inhibition of the action potential firing was clearly evident (Fig 7a–7c). This sequence of light flashes was reproducible with repeated applications (Fig 7b and 7c). Importantly, these experiments were conducted in the cell-attached mode such that the cellular ionic gradients were not changed. Finally, the ability of GABA-B receptor activation to inhibit action potential firing was examined in the presence of both caged compounds in the cell-attached mode (Fig 7d, GABA-A receptors were blocked for this experiment). Activation of GABA-B receptors by blue light induced a pause in spiking while near-UV light increased the firing rate. When both pulses were applied, the increase in spiking rate was blocked.

Taken together, these experiments show that the new caged compounds enable, for the first time, two-color 1P uncaging to be effective in repeated trials evoking bidirectional signaling in a rapid and highly reproducible manner.

## Discussion

Previous development of optically tuned DEAC photochemical protecting groups resulted in the creation of DEAC450, with a red shift of the λ_max_ and a pronounced λ_min_ at the short wavelength where traditional near-UV caging chromophores absorb [21,22]. This resulted in the ability to combine two separate caged compounds (GABA and glutamate) in single experiments with 2P uncaging. The modification introduced here further reduced the λ_min_ in the near-UV range by 50%, enabling two-color 1P uncaging of glutamate and GABA in the same preparation with minimal optical cross-talk. Photolysis of each caged compound was characterized over a wide range of activation powers using two different neuronal preparations. The results confirm that there is a significant power window where each compound, when partnered by its complementary wavelength of light, selectively activates AMPA and GABA-A receptors. The results go on to show the selective activation of two classes of GPCRs, GABA-B and Group I mGlu receptors. Finally, in the presence of both caged compounds, action potentials evoked by activation of AMPA receptors were blocked by co-activation of GABA-A and GABA-B receptors, respectively. This bidirectional modulation of neuronal excitability was highly reproducible without decline in the ability to apply either wavelength in an arbitrary order.

This work is an advance on previous reports of combined and selective 1P photolysis, which unlike the present work, were not truly orthogonal [23] [8,24]. In those experiments each caging chromophore absorbed some light and was therefore photolyzed to a certain degree. Even the best example of two-color photolysis in the chemical literature has this problem [25]. These reports highlight the importance of “read out” for the assessment of two-color optical selectivity. The present investigation points out how differences in receptor affinity and signaling influence the apparent wavelength selectivity of caged compounds. Given the increased affinity and downstream amplification, the activation of GABA-B receptors with near UV light was significantly greater than that for GABA-A receptors (Figs 4 and 5). While, from a chemical point of view, the smallest amounts of photolysis of a long wavelength cage with short wavelength light can be detected with highly sensitive techniques such as NMR or HPLC, the same amount of photolyzed neurotransmitter might not be enough to elicit a significant response in a biological context. Therefore, while the chromatic selectivity might inherently not be zero, it is functionally zero in the biological application.

The key feature of the work described here is that an analytic method was selected that is very sensitive to the amount of activation, thus it allowed the “zero functional threshold” to be defined precisely. Importantly, it can be used for both optical channels when each wavelength produces independent and opposite effects. The noise (or detection limit) for single trials is about 5 pA. Power levels with short wavelength light were established that uncaged glutamate on neurons with no detectable inhibitory signal from caged GABA (Figs 2 and 4). The “power window” for such was easy to establish and, for practical purposes, very wide (100–200 pA of excitation with no inhibition). Thus, in the presence of dcPNPP-Glu, flashes of near-UV light could be applied repeatedly to evoke the generation of action potentials without photolysis of DEAC454-GABA (Fig 6). When flashes of near-UV light were preceded by blue light the action potentials were blocked. The inhibition of action potentials was reversible and repeatable (Figs 6 and 7). The development of DEAC454-GABA therefore enabled real, chromatically-selective uncaging. It should be noted that both compounds reduced the amplitude of mIPSCs at concentrations used for uncaging. Although this might affect the background activity in the slice preparation it did not interfere with the ability of our two-color approach to control excitation and inhibition of the individual cell.

In a previous report of dual 1P uncaging of DEAC450-cAMP and CDNI-GABA, the level of analysis was limited by the effects of the biological receptors controlled by cAMP [14]. The depolarization induced by photolysis of DEAC450-cAMP resulted in a prolonged increase in action potential generation. Photolysis of CDNI-GABA resulted in an inhibition of action potential firing. The prolonged increase in firing induced by DEAC450-cAMP limited an analysis of repetitive optical activation of the two caged compounds. In the present study, the action of two caged neurotransmitters on dopamine neurons allowed repetitive bouts of activation and inhibition such that the power windows for control of membrane potential could be examined using the modulation of spike enhancement and shunting (Fig 7). The rapid nature of both extracellular signals meant that trains of short, long, and long/short wavelength uncaging pulses could be applied repeatedly on single neurons.

The resolution of whole-field 1P uncaging is not suited to stimulate individual synapses resulting in the simultaneous activation of synaptic and extrasynaptic receptors. The temporal activation of separate receptor is an advantage over other forms of exogenous application. These compounds can be used to assess changes in intrinsic excitability of neurons without the complication of changes presynaptic mechanism that control transmitter release. Thus postsynaptic modulation induced by pretreatment of animals with drugs and or behavioral manipulations can be assessed. Spatial precision could be increased by using dual-color, laser-scanning photostimulation with two lasers. The one laser version of this method[1] is a standard technique and is widely used with caged glutamate probes that are photolyzed with near-UV lasers[26,27,28,29,30,31,32], and such systems are already fully compatable with blue lasers. This approach would significantly increase the spatial resolution without sacrificing the optical selectivity which solely depends on the wavelength, not the mode of light delivery.

The use of chromatically-selective 2P-2P photolysis for release of glutamate and GABA in brain slices has been previously established [7,33]. In addition, 2P-1P uncaging of glutamate and GABA has been successfully used in several studies [4,5,6,7]. The present study demonstrated 1P-1P uncaging to activate glutamate and GABA receptors selectively and independently.

## Materials and methods

### General chemical methods

All chemicals were purchased from commercial sources unless otherwise noted. Synthesis and handling of compounds **1**, **3** and **4** were performed under red-filtered light. Reactions were monitored by thin-layer chromatography (TLC) on Merck KGaA glass silica gel plates (60 F_254_) and were visualized with UV light or potassium permanganate staining followed by heating. Flash chromatography was performed using Agela Technologies industrial grade silica (200–300 mesh, 40–60 μm). NMR spectra were recorded on an Oxford 300 MHz NMR spectrometer and the chemical shifts are reported in ppm using the solvent peak as the internal standard. High resolution mass spectral data was obtained at Hunter Collegeon anAgilent iFunnel 6550 Q-ToF mass spectrometer. Hunter Mass Spectrometry is supported by the City University of New York, the National Science Foundation (USA), and the National Institute on Minority Health and Health Disparities (NIH, USA). Analytical HPLC was performed on a Beckman System Gold device with diode array detector using a Grace Altima C18 column (250 mm x 4.6 mm). dcPNPP-Glu was synthesized as previously described [20].

#### Synthesis of dendrimer 2

Propargyl-[G3]-(OH)_8_ bis-MPA dendrimer [12] (0.4005 g, 0.461 mmol, 1.0 equiv) was dissolved in pyridine (10 mL). To this solution was added a solution of *tert*-butyl-hemisuccinate anhydride (1.5834 g, 4.793 mmol, 10.4 equiv) in dichloromethane (30 mL), followed by 4-dimethylaminopyridine (45.0 mg, 0.369 mmol, 0.8 equiv). The reaction mixture was stirred at room temperature for 5 d, after which time dichloromethane (100 mL) was added and the solution was washed with 10% aq. NaHSO_4_ (x2), sat. aq. NaHCO_3_ (x2) and brine, dried over MgSO_4_, filtered and concentrated in vacuo. The crude material was purified by column chromatography (silica, gradient, 20% hexanes/ethyl acetate– 40% hexanes/ethyl acetate), resulting in the isolation of 0.8850 g of dendrimer **2** (91% yield) as a colorless oil. ^1^H NMR (300 MHz, CDCl_3_) δ 4.74 (d, J = 2.5 Hz, 2H), 4.39–4.12 (m, 28H), 2.65–2.47 (m, 33H), 1.43 (s, 72H), 1.31 (s, 3H), 1.25 (s, 6H), 1.23 (s, 12H); ESI-MS m/z calc’d for C_102_H_156_O_46_ [M+Na]^+^ 2139.9765, found 2139.9777.

#### Synthesis of dendrimer-conjugate 4

Dendrimer **2** (0.148 g, 0.07 mmol, 1.0 equiv) and azide **3** [17] (0.0491 g, 0.084 mmol, 1.2 equiv) were dissolved in tetrahydrofuran (6 mL) and water (7 mL). To this mixture was added copper (II) sulfate (5.6 mg, 0.035 mmol, 0.5 equiv) and sodium L-ascorbate (13.9 mg, 0.07 mmol, 1.0 equiv) and the resulting solution was stirred at 50°C under N_2_ atmosphere for 18 h. Water was added and the solution was extracted with dichloromethane (x3), washed with brine, dried over Na_2_SO_4_, filtered and concentrated in vacuo. The crude material was purified by column chromatography (silica, gradient, 30% ethyl acetate/hexanes –ethyl acetate). The product was dissolved in ethyl acetate (10 mL) and filtered through a 0.45 μm PTFE syringe filter and solvent was removed in vacuo, resulting in the isolation of 0.1480 g of dendrimer-conjugate **4** (78% yield) as a yellow oil. ^1^H NMR (300 MHz, CDCl_3_) δ 7.84 (s, 1H), 7.75 (d, J = 15.1 Hz, 1H), 7.50 (d, J = 9.3 Hz, 1H), 7.22 (d, J = 15.6 Hz, 1H), 6.75 (br s, 1H), 6.61 (dd, J = 9.3, 2.6 Hz, 1H), 6.45 (d, J = 2.5 Hz, 1H), 5.37 (s, 2H), 5.23 (s, 2H), 4.98 (br s, 1H), 4.56–4.36 (m, 2H), 4.31–4.07 (m, 28H), 3.57–3.21 (m, 6H), 3.21–2.99 (m, 2H), 2.64–2.44 (m, 32H), 2.37 (t, J = 7.2 Hz, 2H), 2.28–2.09 (m, 2H), 1.79 (p, J = 7.1 Hz, 2H), 1.39 (s, 81H), 1.28–1.12 (m, 27H).; ESI-MS m/z calc’d for C_131_H_196_N_6_O_53_ [M+2H]^2+^ 1351.6486, found 1351.6490.

#### Synthesis of DEAC454-GABA (1)

Dendrimer-conjugate **4** (0.1246 g, 0.046 mmol, 1.0 equiv) was suspended in dichloromethane (2 mL). Trifluoroacetic acid (2 mL) was added and the solution was stirred at room temperature for 2.5 h. The reaction mixture was placed in a 30°C water bath and solvent was removed by bubbling with N_2_ gas. Once the solvent was removed the product was dried in vacuo, resulting in the isolation of 0.0970 g of DEAC454-GABA (**1**, 98% yield) as a yellow semi-solid. ^1^H NMR (300 MHz, CD_3_OD) δ 8.12 (s, 1H), 7.79–7.67 (m, 2H), 7.27 (d, J = 15.2 Hz, 1H), 6.79 (dd, J = 9.3, 2.6 Hz, 1H), 6.55 (d, J = 2.5 Hz, 1H), 5.50 (s, 2H), 5.29 (s, 2H), 4.58–4.46 (m, 2H), 4.38–4.16 (m, 28H), 3.60–3.43 (m, 6H), 3.07–2.95 (m, 2H), 2.69–2.48 (m, 34H), 2.27–2.15 (m, 2H), 1.97 (p, J = 7.2 Hz, 2H), 1.33–1.19 (m, 27H); ESI-MS m/z calc’d for C_94_H_124_N_6_O_51_ [M+2H]^2+^ 1077.3720, found 1077.3726.

#### DEAC454-GABA photolysis quantum yield

The quantum yield of photolysis was determined by comparative photolysis of DEAC454-GABA and DEAC450-Glu [34]. A solution was prepared in pH 7.4 HEPES buffer containing DEAC454-GABA and DEAC450-Glu with equivalent optical densities at 405 nm and with inosine as a photochemically inert internal standard [34]. The solution was placed in a 1 mm cuvette and photolyzed using a defocused 405 nm laser with a diameter of 3 cm. HPLC was used to determine the percentage of compound photolysis over time and the relative rates of photolysis were compared.

### Animal care and use

All experiments were approved by institutional IACUC.

### Slice preparations

For experiments on dopamine neurons, male and female Sprague-Dawley rats (6–10 weeks) were anesthetized with isoflurane, killed by decapitation and the brain removed. The brain was sliced at 220 μm in horizontal orientation in warm artificial cerebrospinal fluid (ACSF) containing (in mM): 126 NaCl, 2.5 KCl, 1.2 MgCl_2_, 2.6 CaCl_2_, 1.2 NaH_2_PO_4_, 11 D-glucose, 21.4 NaHCO_3_, and 0.03 MK801 (5*S*,10*R*)-(+)-5-methyl-10,11-dihydro-5*H*-dibenzo[a,d]cyclohepten-5,10-imine (Abcam, Cambridge, UK), equilibrated with 95% O_2_/5% CO_2_. Slices were allowed to recover at 34°C for at least 30 minutes with oxygenated (95% O_2_/ 5% CO_2_) ACSF containing (in mM): 126 NaCl, 2.5 KCl, 1.2 MgCl_2_, 2.6 CaCl_2_, 1.2 NaH_2_PO_4_, 11 D-glucose, 21.4 NaHCO_3_ and 0.4 MK801. For experiments on hippocampal CA1 neurons male and female C57BL/6J mice (3–10 weeks) were anesthetized with isoflurane, killed and the brain removed. The brain was cut in horizontal orientation into 350 μm thick slices in ice-cold cutting solution containing (in mM): 60 NaCl, 2.5 KCl, 1.25 NaH_2_PO_4_, 7 MgCl_2_, 0.5 CaCl_2_, 26 NaHCO_3_, 10 glucose, 100 sucrose, equilibrated with 95% O_2_/5% CO_2_. The sections were stored for 15 minutes at 33°C in the same solution and then transferred to ACSF at room temperature containing (in mM): 125 NaCl, 2.5 KCl, 1.25 NaH_2_PO_4_, 1 MgCl_2_, 2 CaCl_2_, 26 NaHCO_3_, 10 glucose (95% O_2_/ 5% CO_2_).

### Electrophysiology recordings

For experiments on dopamine neurons, after incubation, slices were hemisected and transferred to the recording chamber and superfused with ACSF (1.5 mL/minute, 34°C). Dopamine neurons were identified by their location (rostrolateral of the medial terminal nucleus of the accessory optic tract). The identity of dopamine neurons was further validated by observing the characteristic pacemaker spiking of the neuron in cell-attached mode with an action potential duration of >2 ms. Whole-cell recordings were made with an Axopatch 1D amplifier (Molecular Devices, Sunnyvale, CA, USA) in voltage-clamp mode (*V*_hold_ = −60 mV). Recording pipettes (1.7–2.1 MΩ) were filled with internal solution containing (in mM): 60 potassium methanesulfonate, 55 KCl, 20 NaCl, 1.5 MgCl_2_, 5 HEPES(K), 0.1 EGTA, 2 Mg-ATP, 0.25 Na-GTP, pH 7.4, 280–285 mOsM. Series resistance was monitored without compensation and was <15 MΩ. Current was continuously recorded at 200 Hz with PowerLab (chart version 5.4.2; AD Instruments, Colorado Springs, CO). Episodic currents were recorded at 10 kHz for 1 minute using AxoGraphX (1.4.3; AxographX, Berkeley, CA). Drugs were applied by bath superfusion. Cell-attached recordings were made with recording pipettes (2–2.5 MΩ) filled with ACSF.

For experiments on hippocampal CA1 neurons, slices were transferred to the recording chamber and superfused with ACSF at room temperature. Whole-cell recordings were made with an EPC-9 amplifier (HEKA Instruments, Bellmore, NY) in voltage- or current-clamp mode by using one of the following internal solutions. Glutamate currents in Fig 2 (voltage clamp *V*_hold_ = −60 mV) and potentials in Figs 3 and 7 (current clamp, *V*_m_ adjusted to ~-60 mV by constant current injection) were recorded with (in mM): 135 K-gluconate, 4 MgCl_2_, 10 HEPES, 5 EGTA, 4 Na_2_-ATP, 0.4 Na_2_-GTP, 10 Na_2_ phosphocreatine, pH 7.35 (calculated AMPA receptor reversal potential: -1 mV). GABA currents in Fig 2 (*V*_hold_ = +10 mV) and mIPSCs in Fig 1d (*V*_hold_ = -70 mV) were recorded with the same solution as above but replacing K-gluconate with equimolar concentration of Cs-methanesulfonate (calculated Cl^-^ reversal potential: -71 mV) or CsCl_2_ (calculated Cl^-^ reversal potential: +2 mV), respectively. Current or voltage were recorded at 20 kHz and filtered at 3 kHz with Patchmaster/Pulse (HEKA).

The uncaging experiments were carried out in the dark with a very simple and inexpensive setup that employed LEDs along with the appropriate emission filters mounted on the fluorescence port of the microscope. Uncaging of DEAC454-GABA (25–30 μM) and dcPNPP-Glu (200–350 μM) was carried out with full-field illumination (365 and 450 or 470 nm LED, Thorlabs, NJ, USA) coupled through a 60x objective (Olympus, 0.9 numerical aperture). The LEDs were mounted on the epifluorescence port of a BX51 or 61 microscope (Olympus). The beams were combined with a long-pass dichroic (387nm, Chroma, Bellows Falls, VT, USA). For experiments on dopamine neurons, a notch filter (365/20nm, Chroma) was placed in the light path from the 365 nm LED. The 450 nm LED was filtered in all recordings (455/20nm notch filter, Chroma). A long-pass dichroic (488 nm, Semrock, Rochester, NY, USA) in the fluorescence turret was used to direct the beams to the objective. Light power and duration was controlled via the LED driver (LEDD1B, Thorlabs) by external voltage modulation. The power was measured with a photometer (S120VC, Thorlabs) prior to the experiment. Solutions containing either DEAC454-GABA, dcPNPP-Glu, or both (4–7 mL) were recirculated for at least 5 minutes before uncaging. Blocker and antagonists were purchased from Tocris (Minneapolis, MN, USA) or Sigma-Aldrich (St. Louis, MO, USA) and applied via the perfusion system. The specific blocker/antagonists used for each experiment are given in the respective figure legends.

To determine the spike threshold (Fig 3), increasing current injections were applied in the current clamp mode (2–5 ms, 100 pA steps). Current injections eliciting spikes were then paired with 470 nm or 365 nm uncaging pulses in the presence of DEAC454-GABA of varying energy and duration to determine the spike block threshold, respectively. After each set of recordings, the spike threshold was reconfirmed and cells discarded in case it shifted.

### Data analysis

Analysis of power/duration/current relationships (Fig 2) was carried out using FitMaster (HEKA), Excel (Microsoft, Redmond, WA) and IGOR Pro (WaveMetrics, Lake Oswego, OR). mIPSCs (Fig 1c) were detected using the template search of pClamp (Molecular Devices, Sunnyvale, CA). The isolated events were then analyzed using custom-written IGOR Pro procedures (WaveMetrics).

### Statistics

Data are presented as mean +/- SEM. The average value for each individual cell was used for statistical analysis.

Data in Fig 1c were tested for normal distribution with the Kolmogorov-Smirnov test. In Fig 1c, mIPSC amplitudes were tested by a One-Way ANOVA with post-hoc Tukey Test while mIPSC frequencies were tested with a modified One-Way ANOVA (using the log of the data) since the variances were significantly different. Data in Fig 7 were tested with a repeated measures Two-Way ANOVA with post-hoc Tukey Test to examine multiple comparisons. Significance level was set to p < 0.05 if not stated otherwise. Statistical testing was carried out using Prism (GraphPad, La Jolla, CA) and Excel (Microsoft).
